# Non-ST Elevation Myocardial Infarction in the Elderly. Antithrombotic Therapy and Beyond

**DOI:** 10.31083/j.rcm2407201

**Published:** 2023-07-13

**Authors:** Pablo Díez-Villanueva, César Jiménez-Méndez, José Luis Ferreiro, Pedro Cepas-Guillén, Clara Bonanad, Sergio García-Blas, Albert Ariza-Solé, Juan Sanchís, Manuel Martínez-Sellés

**Affiliations:** ^1^Cardiology Department, Hospital Universitario La Princesa, 28006 Madrid, Spain; ^2^Cardiology Department, Hospital Universitario Puerta del Mar, 11009 Cádiz, Spain; ^3^Department of Cardiology, Bellvitge University Hospital, 08907 L’Hospitalet de Llobregat, CIBERCV, Spain; ^4^Bio-Heart Cardiovascular Diseases Research Group, Bellvitge Biomedical Research Institute (IDIBELL), 08907 L’Hospitalet de Llobregat, Spain; ^5^Cardiology Department, Hospital Clinic, 08036 Barcelona, Spain; ^6^Cardiology Department, Hospital Clínico Universitario, 46010 Valencia, Spain; ^7^Instituto de Investigación Sanitaria INCLIVA (JR/21/00041), 46010 Valencia, Spain; ^8^Department of Medicine, Faculty of Medicine, University of Valencia, 46010 Valencia, Spain; ^9^Cardiology Department, Hospital Clínico Universitario, 46010 Valencia, CIBERCV, Spain; ^10^Cardiology Department, Hospital General Universitario Gregorio Marañón, 28007 Madrid, CIBERCV, Spain; ^11^Universidad Europea de Madrid, 28670 Madrid, Spain; ^12^Universidad Complutense de Madrid, 28007 Madrid, Spain

**Keywords:** elderly, acute coronary syndrome, non-ST segment elevation myocardial infarction, antithrombotic therapy, frailty

## Abstract

Non-ST segment elevation myocardial infarction (NSTEMI) is the most frequent type of acute coronary syndrome in the elderly. Antithrombotic therapy is the cornerstone of pharmacological therapy in the setting of an acute ischemic event, a clinical scenario in which thrombotic and bleeding risks ought to be considered, particularly in older patients. In this article, specific aspects of antithrombotic therapy in elderly patients with NSTEMI are reviewed, including pharmacokinetic and pharmacodynamic characteristics and different clinical situations. The role of frailty and other common geriatric conditions, that are associated with worse prognosis in elderly patients with cardiovascular disease, is also addressed.

## 1. Introduction

**Fig. 1.**
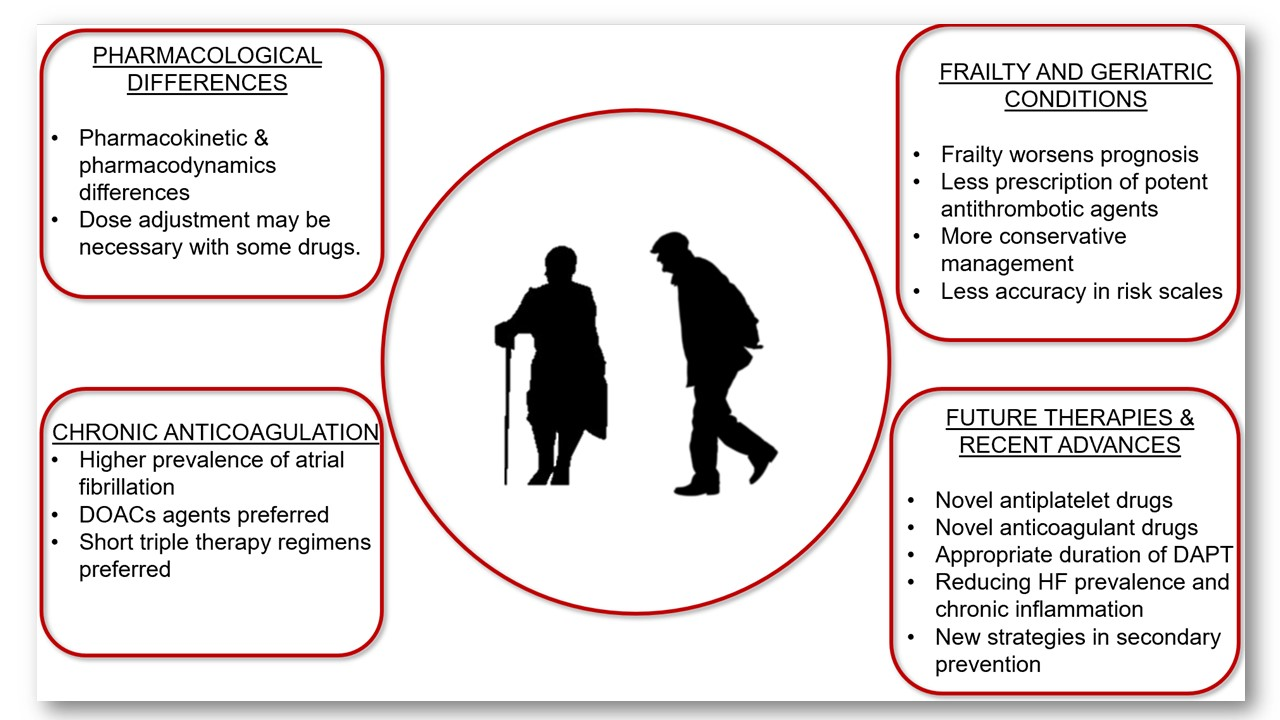
**Update on antithrombotic therapy in the elderly with non-ST segment elevation acute myocardial infarction**. DOACs, direct oral anticoagulant agents; DAPT, dual antiplatelet therapy; HF, heart failure.

Non-ST segment elevation myocardial infarction (NSTEMI) is the most frequent type of acute coronary syndrome (ACS) in older patients, those over 75 years old, who constitute the main focus of this review [1, 2]. Antithrombotic therapy is the cornerstone of pharmacological therapy in patients with ACS since it is associated with a significant reduction in ischemic events, although at the expense of an increased risk of bleeding, especially in the elderly [1, 2]. In this setting, and according to current recommendations, a comprehensive assessment of ischemic and hemorrhagic risks should be carefully performed. This is a complex clinical scenario, in which it is important to assess and consider the role and prognostic impact of comorbidities and geriatric conditions, common in the elderly population. Fig. 1 summarizes the main concepts addressed in the text.

## 2. Pharmacology of Antithrombotic Agents: Focus on the Elderly

The risk of atherothrombotic and bleeding events is higher in elderly patients with ACS compared to younger subjects, which poses important challenges at the time of selecting the most appropriate antithrombotic regimens [3, 4]. In addition, pharmacological responsiveness, clinical efficacy and drug interactions might be altered by age-related conditions such as hemostatic alterations (decreased fibrinolysis, increased clotting, endothelial dysfunction, heightened platelet reactivity), changes in pharmacokinetics (diminished absorption, alterations in hepatic metabolism—e.g., reduced cytochrome P450 (CYP) activity-, changes in renal clearance leading to reduced elimination…), comorbidities and polypharmacy (increasing the risk of drug interactions) [5].

The most frequently used oral antiplatelet agents in ACS patients are aspirin and ${\mathrm{P2Y}}_{12}$ antagonists (clopidogrel, prasugrel, and ticagrelor), either alone or in combination as dual antiplatelet therapy (Table 1).

**Table 1.** **Pharmacological properties of the most commonly used oral antiplatelet agents in acute coronary syndromes**.

	Aspirin	Clopidogrel	Prasugrel	Ticagrelor
Group	COX-1 inhibitor	${\mathrm{P2Y}}_{12}$ inhibitor: thienopyridine	${\mathrm{P2Y}}_{12}$ inhibitor: thienopyridine	${\mathrm{P2Y}}_{12}$ inhibitor: CPTP
Direct action	Yes	No	No	Yes*
Receptor blockade	Irreversible	Irreversible	Irreversible	Reversible
Onset of action**	15–30min	2–6 h	30 min–4 h	30 min–2 h
Offset of action	3–7 days	5–10 days	7–10 days	3–5 days
Drug interactions (CYP system)	No	Yes	No	Yes
Considerations in elderly patients	No	Increased rate of poor response	If ≥75 years, dose reduction to 5mg o.d.	No

COX, cyclooxygenase; CPTP, cyclopentyltriazolopyrimidine; CYP, cytochrome P450. *Although ticagrelor is direct-acting, approximately 30–40% of its antiplatelet effectiveness is due to an active metabolite (AR-C124910XX). **It may vary according to clinical setting.

Aspirin is an irreversible inhibitor of the platelet cyclooxygenase-1 enzyme with a potentially increased pharmacodynamic sensitivity in older patients, although this phenomenon has not been fully established [6, 7]. Of note, the higher vulnerability of older patients to gastrointestinal disturbances (due sometimes to chronic abuse of non-steroidal anti-inflammatory agents) may prompt physicians to pay a special attention to elderly patients receiving antiplatelet therapy in order to prevent gastrointestinal bleeding. Clopidogrel is still the most widely used ${\mathrm{P2Y}}_{12}$ inhibitor in older patients probably due to its lower risk of bleeding compared to prasugrel or ticagrelor [8]. However, clopidogrel has broad variability in response and the prevalence of poor responsiveness is higher among older subjects [9]. On the other hand, clopidogrel appears to be a reasonable alternative to ticagrelor in NSTEMI elderly patients ≥70 years according to the individual ischemic and bleeding risk profile, since it was associated with lesser bleeding rates without an increase in the combined endpoint of all-cause death, myocardial infarction, stroke and bleeding in the POPular AGE trial [10].

With regards to prasugrel, an increasingly higher risk of bleeding among patients over 75 years was found in TRITON-TIMI 38 trial based on a regimen of prasugrel 10 mg once daily (o.d.). This effect has in part been attributed to an augmented exposure to its active metabolite [11, 12]. Then, a reduced dose of prasugrel (5 mg o.d.) is recommended in patients ≥75 years of age, which has a slightly more potent platelet inhibitory effect compared to clopidogrel [13]. However, the clinical benefit of this regimen has not been well proven yet. In particular, the ELDERLY ACS-2 trial investigated the clinical efficacy of half-dose prasugrel (5 mg o-d) in an elderly ACS population (≥74 years). Of note, this trial was stopped prematurely due to futility as the half-dose prasugrel regimen was not superior to regular-dose clopidogrel, showing no differences in ischemic events but a numerically higher rate of bleeding events [14]. Conversely, no dose adjustment because of age is needed for ticagrelor as the benefits of this drug compared to clopidogrel were maintained in all age subgroups in the pivotal PLATO trial [15].

Finally, platelet function tests have been proposed as a tool to guide antithrombotic therapy adjustments in high-risk bleeding groups such as elderly patients after an ACS. However, in a randomized clinical trial, a platelet function monitoring strategy was not associated with fewer bleeding or ischemic events in this subgroup [16].

Currently, there are two groups of parenteral antiplatelet agents available in ACS patients: glycoprotein IIb/IIIa inhibitors (GPIs) and cangrelor, a ${\mathrm{P2Y}}_{12}$ receptor antagonist (Table 2). These are used almost exclusively in the setting of percutaneous coronary intervention (PCI). GPIs are the most potent antiplatelet agents in the armamentarium, among them tirofiban and eptifibatide are currently available for clinical use. Noteworthy, both drugs require dose adjustment in patients with impaired renal function [3]. No specific dose-adjustment for GPIs according exclusively to age is recommended, although careful selection of patients is mandatory because excessive in bleeding rates with GPIs have been observed in older patients [17]. Cangrelor, an intravenous ${\mathrm{P2Y}}_{12}$ inhibitor, has no hepatic or renal metabolism and a short half-life so no age-related pharmacological issues have been reported to date [18].

**Table 2.** **Pharmacological properties of currently approved parenteral antiplatelet agents**.

	Cangrelor	Abciximab	Tirofiban	Eptifibatide
Group	${\mathrm{P2Y}}_{12}$ inhibitor	GPI	GPI	GPI
Molecular structure	ATP analog	Fab of a monoclonal antibody	Non-peptide synthetic molecule	Synthetic cyclic heptapeptide
Reversibility	Yes	Yes*	Yes	Yes
Plasmatic half-life	3–6 min	Biphasic: <10 min and ∼30 min	∼2 hours	∼2.5 hours
Duration of antiplatelet effect after discontinuation	60–90 min	Platelet life-span	∼4–8 horas	∼4 horas
Renal adjustment	No	No	Reduce infusion by 50% if CrCl <30 mL/min	Reduce infusion by 50% if CrCl 30–50 mL/min
		Contraindicated in hemodialysis		Contraindicated if CrCl <30 mL/min or hemodialysis
Considerations in elderly patients	No	Caution due to increased bleeding risk	Caution due to increased bleeding risk	Caution due to increased bleeding risk

ATP, adenosine triphosphate; CrCl, creatinine clearance; Fab, antigen-binding fragment; GPI, glycoprotein IIb/IIIa inhibitor. *Frequently reported as an irreversible agent due to its great affinity for the receptor.

Parenteral anticoagulation is also an important part of the initial therapeutic management of NSTEMI patients, especially those undergoing PCI [1]. The pharmacological features of the most frequently used parenteral anticoagulant agents are presented in Table 3. Unfractionated heparin is the only agent that can be used in patients with severe renal impairment, while enoxaparin (a low-molecular-weight heparin) and bivalirudin (a direct thrombin-inhibitor) require dose adjustment below certain levels of creatinine clearance (CrCl). Fondaparinux is contraindicated if CrCl <20 mL/min. Importantly, a reduction in subcutaneous dosing of enoxaparin for patients aged ≥75 years from 1 to 0.75 mg/kg (without initial bolus) is recommended, whereas the other parenteral anticoagulant agents do not require a specific age-related dose adjustment [3, 5].

**Table 3.** **Pharmacological properties of parenteral anticoagulant agents used in acute coronary syndromes**.

	Unfractionated heparin (UFH)	Enoxaparin	Fondaparinux	Bivalirudin
Target	IIa and Xa (IXa, XIa and XIIa to a lesser extent)	Xa (IIa to a lesser extent)	Xa	IIa
Route of administration	IV	IV, SC	SC	IV
Direct action	No*	No*	No*	Yes
Plasmatic half-life	60–90 min	4–5 h (SC)	25 min	25 min
Reversal agent	Protamine	Protamine (partial reversal: 40–70%)	No	No
Renal adjustment	No	Dose reduction if CrCl <30 mL/min	Contraindicated if CrCl <20 mL/min	Reduce infusion if CrCl 30–59 mL/min
		Contraindicated if CrCl <15 mL/min		Contraindicated if CrCl <30 mL/min
Considerations in elderly patients	No	If ≥75 years, dose reduction to 0.75 mg/kg b.i.d. and no initial bolus	No	No

CrCl, creatinine clearance; IV, intravenous; SC, subcutaneous. *Need a cofactor: antithrombin III.

## 3. Antithrombotic Treatment in an Elderly Patient with ACS and Indication for Oral Anticoagulation

Atrial fibrillation (AF) affects up to one third of patients with coronary artery disease, especially in the elderly [1]. In elderly patients with AF, anticoagulation is indicated, with direct oral anticoagulants (DOACs) constituting the treatment of choice [19, 20]. Table 4 summarizes standard doses for DOACs. When specific criteria are present, adjusted doses must be prescribed.

**Table 4.** **Direct-acting anticoagulants standard and adjusted doses**.

	Apixaban	Edoxaban	Dabigatran	Rivaroxaban
Standard doses	5 mg twice daily	60 mg once daily	150 mg twice daily	20 mg once daily
Reduced doses	2.5 mg twice daily if two or more criteria*	30 mg once daily if	110 mg twice daily	15 mg once daily
	Age ≥80 years	Weight <60 kg	Age >80 years	ClCr <50 mL/min *
	Weight <60 kg	ClCr <50 mL/min *		
	Creatinine ≥1.5 mg/dL	Concomitant use of P-gp inhibitors		
	*Also if ClCr <30 mL/min			

* According to Cockroft-Gault formula. ClCr, Creatinine clearance; P-gp, glycoprotein P.

However, a high percentage of elderly patients with AF do not receive or receive inadequate doses of anticoagulant therapies. This inappropriate use of a reduced dose has been associated with age itself, concomitant use of antiplatelet therapy, subjective perceptions of the physician and the overestimation of the bleeding risk [21, 22, 23, 24]. The results of DOACs trials in patients with indication (indications) of chronic anticoagulation and dual antiplatelet therapy are summarized in Table 5. When DOACs cannot be used (e.g., mechanical prosthetic valves) vitamin K antagonists plus antiplatelet regimens based on clopidogrel is indicated [1, 25]. According to current guidelines, it is recommended to shorten the triple therapy (anticoagulant + aspirin + clopidogrel) as much as possible, from 1 week to 1 month depending on patient’s risk profile [1, 26, 27, 28, 29].

**Table 5.** **Main pivotal trials including patients with acute coronary syndrome and indication of anticoagulation with direct oral anticoagulants**.

Clinical trial	Description	Primary outcome	Results
PIONEER AF-PCI	2124 patients. Safety comparison of 2 strategies of rivaroxaban (2.5 mg/15 mg) + DAPT vs triple therapy 12 months	Clinically relevant bleeding TIMI (safety)	Rivaroxaban therapy was associated with a significant decrease in bleeding compared to triple VKA therapy.
REDUAL-PCI	2725 patients. Safety of 2 doses of dabigatran (110 mg/12 h and 150 mg/12 h) vs standard triple VKA therapy 24 months	Clinically relevant bleeding ISTH (safety)	Dual therapy with dabigatran (with both doses) is safer than using triple therapy.
AUGUSTUS	4600 patients. Non-inferiority trial of Apixaban vs VKA combined with ${\mathrm{P2Y}}_{12}$ inhibitor. To demonstrate superiority of single antiplatelet therapy with aspirin vs placebo. 6 months.	Clinically relevant bleeding ISTH (safety)	The rate of bleeding was lower with Apixaban than with VKA in triple therapy; in addition, a reduction in bleeding was observed with placebo vs Aspirin.
ENTRUST AF-PCI	1506 patients. Safety of Edoxaban + ${\mathrm{P2Y}}_{12}$ vs triple therapy with VKA + ${\mathrm{P2Y}}_{12}$ + Aspirin 12 months.	Clinically relevant bleeding ISTH (safety)	Edoxaban demonstrated non-inferiority for bleeding compared to VKA therapy.

DAPT, dual antiplatelet therapy; VKA, vitamin K antagonists; TIMI, Thrombolysis in Myocardial Infarction; ISTH, International Society on Thrombosis and Haemostasis.

## 4. Role of Frailty, Comorbidity and Other Ageing Related Variables on Risk Prediction and Antithrombotic Management in Elderly Patients with NSTEMI

**Fig. 2.**
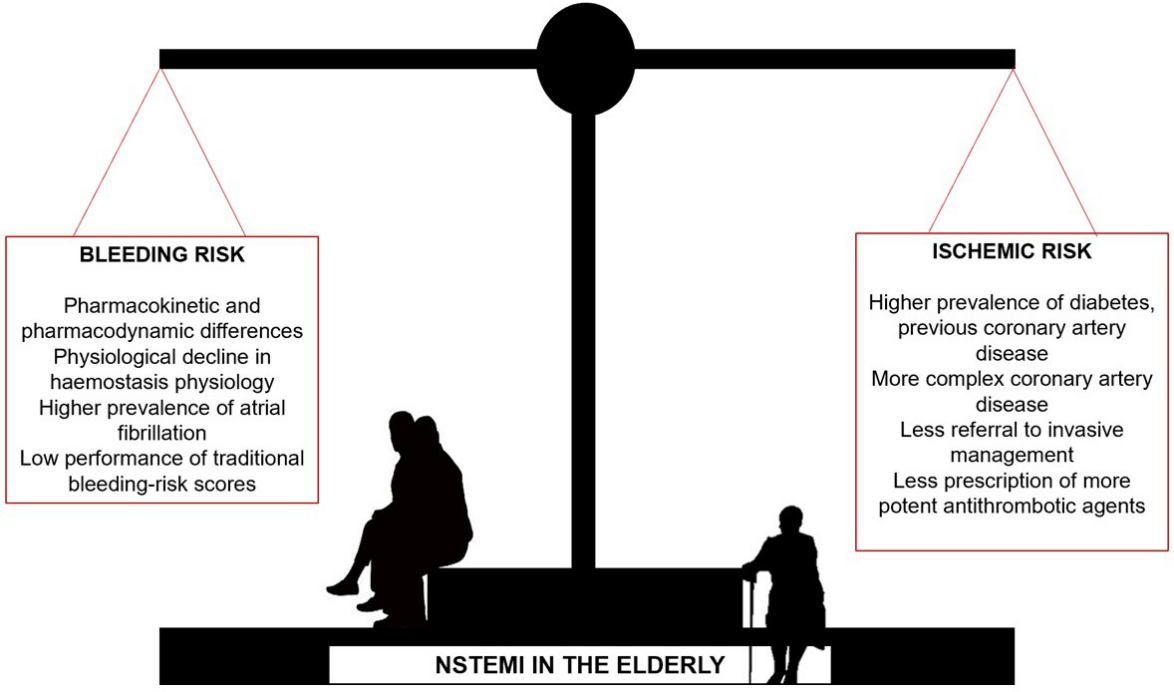
**Ischemic and bleeding risks in the elderly population with non-ST segment elevation acute myocardial infarction**.

Dual antiplatelet therapy (DAPT) is recommended for 12 months after an ACS but duration may vary depending on ischemic and bleeding risks, comorbidities, or need for chronic anticoagulation [1, 27]. Thus, an individualized approach on a case-to-case basis is crucial in order to provide the optimal antithrombotic strategy and its duration for each patient [2]. In this setting, the assessment of comorbidities and a comprehensive geriatric assessment is also of great importance [30]. Besides, most recommended bleeding risk stratification tools come from studies where patients at older ages are clearly underrepresented [31, 32]. The PRECISE DAPT score is recommended for guiding intensity and duration of DAPT after an ACS [1]. However, patients older than 75 years (more than 90%) are frequently at the highest risk category because age accounts for more than half of points needed for reaching this high-risk category. In addition, most of these patients are at high risk for ischemic events, since the prevalence of diabetes, multivessel disease or prior coronary intervention is high. Therefore, the use of this tool has been suggested to be adapted with different thresholds in older patients [24]. On the other hand, and according to the *Academic Research Consortium*, age ≥75 years, but also frequent comorbidities in the elderly like anemia or chronic kidney disease (CKD), constitutes a minor risk factor for bleeding risk. Of note, in this scale high bleeding risk is defined when at least 1 major or 2 minor factors are present [3]. Fig. 2 outlines the complex balance of ischemic and bleeding risks in this population.

Frailty is an age-associated clinical syndrome characterized by a decrease in physiological reserve that entails an increased vulnerability to stressors. In cardiovascular disease, frailty has been associated with worse clinical outcomes and higher morbidity and mortality in all clinical scenarios, in both acute and chronic settings [33]. The awareness of its importance has led to the statement of specific recommendations in order to achieve a prompt identification. In elderly with ACS, the FRAIL scale (Table 6) has been shown to identify patients with worse prognosis [33]. The role of frailty, comorbidity and other geriatric assessment for predicting bleeding risk in patients with ACS has also been assessed, with several studies showing an association between frailty and higher incidence of bleeding [34, 35]. On the other hand, data from the LONGEVO-SCA registry show that only comorbidity according to Charlson index was significantly associated with bleeding, while the contribution of the geriatric assessment was modest [36, 37]. Likewise, a substudy from the FRASER registry found that frailty did not improve the prediction of bleeding over the PRECISE DAPT and PARIS bleeding risk scores [38].

**Table 6.** **FRAIL Scale (Frailty if 3 or more of the following 5 criteria are present)**.

Item	Assessment
Fatigue	Do you feel tired most of the time?
Resistance	By yourself and not using aids, do you have any difficulty walking up a flight of stairs without resting?
Ambulation	By yourself and not using aids, do you have any difficulty walking 100 m?
At least 5 of the following symptoms	Arthritis, diabetes, angina/infarction, hypertension, stroke, asthma, chronic bronchitis, emphysema, osteoporosis, colorectal cancer, skin cancer, depression and anxiety, dementia, leg ulcers
Weight loss	Weight loss >5% in the past year

Some comorbidities such as CKD, or anemia are frequent in this population, associating higher rates of morbidity and mortality, and lesser referral to invasive management [30, 39]. Besides, CKD is a well identified risk factor for development of contrast-induced acute kidney injury in patients undergoing invasive approach [2, 30]. Cancer is the second leading cause of mortality in these patients, and shares common risk factors with coronary artery disease. Its prevalence is higher in the elderly and adversely impacts prognosis. Therefore, a multidisciplinary approach is of great importance [40].

Gender differences have also been identified in the management and prognosis of elderly patients with ACS. Women are more likely to be older, frailer and with higher burden of cardiovascular risk factors [30, 41]. Atypical presentation and delayed diagnosis are also more frequent, and women are treated conservatively more often than men [41]. Moreover, in octogenarians with NSTEMI, female sex has been associated with worse short-term prognosis [42].

Finally, ethical considerations due to ageism may arise in a progressively elderly, more comorbid ACS population. Thus, it is essential to carefully choose best clinical management, also considering patient’s preferences and goals [43].

## 5. Frailty and Invasive Strategy in Elderly Patients with ACS

Age itself is not a contraindication for PCI, since PCI has demonstrated to significantly decrease short and long-term mortality and morbidity in elderly patients with STEMI [2, 26].

However, decision-making is far more complex in NSTEMI. It is well-known that frailty worsens the prognosis [30, 32, 44, 45, 46, 47, 48, 49, 50]. However, whether this means that frail patients should be managed differently remains unknown.

The European guidelines on NSTEMI recommend for older people the same diagnostic and intervention strategies as for younger patients, including radial approach in PCI and the use of drug eluting stents [1]. In this regard, the implantation of a latest-generation drug-eluting stent might allow for the indication of short periods of dual antiplatelet therapy. So, age should not be a limitation. However, age is only the tip of the iceberg. Geriatric conditions, such as comorbidities and frailty, also play a role in the prognosis. Indeed, the guidelines recognize the absence of robust data on the management of frail patients and recommend an individual approach, balancing the potential benefit of treatments with their potential harm. The critical question is: Can an invasive strategy modify the poor prognosis that frailty confers?

The “After-eighty” trial compared invasive vs conservative strategies in patients older than 80 [51]. It should be noted that this study only focused on age. Comorbidities and frailty were not evaluated and were probably underrepresented. On the other hand, no patient received a coronary angiogram regardless of the clinical course in the conservative group. This management is very far from usual clinical practice. The results largely favored the invasive group for a composite endpoint (including death, myocardial infarction, urgent revascularization or stroke). The MOSCA trial meant a step further because it focused on age and comorbidities [52]. The inclusion criteria were NSTEMI, age older than 70, and at least two comorbidities. Patients were randomized to the invasive or conservative strategies in the conservative arm, but a coronary angiogram was permitted if recurrent ischemia. There were no differences for a primary endpoint consisting of the composite of death, reinfarction or readmission for a cardiac cause. However, the study was unpowered since it did not reach the estimated sample. In line with these results, registries of elderly patients with NSTEMI suggest that comorbidities and disability impair the potential benefit of in-hospital revascularization on outcomes [53, 54]. The management (invasive or not) of frail elderly patients with NSTEMI has also been addressed in different registries [55, 56]. The MOSCA-FRAIL clinical trial is the first trial including elderly (age older than 70 years) and frail (defined by four or more points in the Clinical Frailty Scale) patients with NSTEMI [57], randomized to invasive or conservative strategies (with crossover permitted if recurrent ischemia). The primary endpoint was the number of days alive out of hospital during the first year. Interestingly, invasive treatment in frail older patients was not associated with survival benefits [58].

Table 7 (Ref. [51, 52, 58]) summarizes the main characteristics of the clinical trials assessing the impact of an invasive strategy in elderly patients with NSTEMI.

**Table 7.** **Main clinical trial assessing the impact of an invasive management in elderly patients with NSTEMI**.

Study	Population (n)	Mean age (years)	Female sex (%)	Endpoint	Follow up	Frailty and comorbidity assessment
Tegn *et al*. [51]	457	84.8	51%	A composite of death, myocardial infarction, urgent revascularization or stroke	1.5 years	No assessment
After Eighty (2016) [51]						
Sanchis *et al*. [52]	106	82	47%	Death, reinfarction, or readmission for cardiac causes.	2.5 years	No frailty assessment.
MOSCA (2016)						Charlson comorbidity index
Sanchis *et al*. [58]	167	86	38%	Number of days alive out of the hospital from discharge to 1 year follow-up.	1 year	Clinical Frailty
MOSCA-FRAIL						Scale score ≥4
						Comorbidity burden
						Polypharmacy

NSTEMI, non-ST segment elevation myocardial infarction.

## 6. Recent Advances and Future Directions in Antithrombotic Treatment

Although the death rates among elderly patients with ACS have declined significantly, considerable opportunities for improvement remain. Table 8 summarizes the recent advances and their potential benefit in elderly patients with ACS.

**Table 8.** **Recent pharmacologic advances in cardiovascular field and potential cardiovascular benefits in the elderly**.

	Drug Class	Mechanism	Potential cardiovascular benefits in the elderly
Selatogrel	Antiplatelet sc	Reversibly-binding ${\mathrm{P2Y}}_{12}$ inhibitor	Potential early, pre-hospital treatment in ACS, ↓ risk bleeding
Anfibatide	Antiplatelet iv	Platelet receptor GP Ib inhibitor	Antiplatelet effect without ↑ bleeding and thrombocytopenia
Asundexian	Anticoagulant	Oral activated coagulation factor XIa inhibitor	↓ Bleeding compared with apixaban in AF patients
Cardiovascular polypill	Aspirin + ACE inhibitor + statin	Multiple	↑ adherence →↓ CV death, MI, stroke or urgent revascularization

ACE, angiotensin-converting enzyme; ACS, acute coronary syndromes; AF, atrial fibrillation; CV, cardiovascular; GP, glycoprotein; iv, intravenous; MI, myocardial infarction; sc, subcutaneous. ↑: increase. ↓: reduce.

Novel antiplatelet drugs have been recently developed; some explore new pharmacological targets, while others seek to refine existing drugs. Selatogrel (ClinicalTrials.gov Identifier: NCT04957719) is a novel subcutaneous reversibly-binding ${\mathrm{P2Y}}_{12}$ inhibitor, that is being tested in phase III studies. However, its future use in elderly patients is uncertain [59]. The appropriate duration of DAPT after PCI is currently under research. Several studies have suggested that a regimen based one-month of DAPT after PCI may mitigate bleeding risk without compromising safety, compared with longer durations [60, 61]. Due to the excess of bleeding risk in the ACS elderly population, this population could benefit most from this approach.

Bleeding events are related to adverse outcomes in elderly patients with ACS. Reducing the bleeding rates, especially in patients under chronic anticoagulation, may significantly impact on survival [62].

Asundexian, a recently introduced oral anticoagulant agent that inhibits the activated coagulation factor Xia, might reduce thrombosis with minimal effect on haemostasis. Although larger clinical trials are needed, its promising results with very low rates of bleeding, are encouraging and could lead to improve prognosis of elderly patients with ACS and AF [63, 64].

In lights to decrease the high rates of recurrent ischemic events, patient adherence to secondary prevention treatment is crucial. However, adherence rates in the elderly population are low due to the impact of comorbidities or polypharmacy, among others. The SECURE trial showed that in elderly patients admitted for ACS, the use of the cardiovascular polypill was associated with a significantly lower risk of major adverse cardiovascular events [65]. Lastly, a new generation of cholesterol-lowering drugs based on genetic transcription such as inclisiran may reduce the burden of cardiovascular disease [66].

Cardiac rehabilitation programs are essential after an ACS, especially in frail elderly patients, in whom a multidimensional approach is encouraged, aimed to decrease frailty and physical disability [67].

## 7. Conclusions

The management of NSTEMI in the elderly still represents a clinical challenge. Several factors, including frailty and comorbidities, which adversely impact prognosis, must be taken into account when choosing the correct antithrombotic therapy and referring to invasive management. Although new therapies are been tested with potential cardiovascular benefits in the elderly, randomized clinical trials specifically focused on this population are needed.

## References

[1] Collet JP, Thiele H, Barbato E, Barthélémy O, Bauersachs J, Bhatt DL (2021). 2020 ESC Guidelines for the management of acute coronary syndromes in patients presenting without persistent ST-segment elevation: The Task Force for the management of acute coronary syndromes in patients presenting without persistent ST-segment elevation of the European Society of Cardiology (ESC). *European Heart Journal*.

[2] Jiménez-Méndez C, Díez-Villanueva P, Alfonso F (2021). Non-ST segment elevation myocardial infarction in the elderly. *Reviews in Cardiovascular Medicine*.

[3] Andreotti F, Rocca B, Husted S, Ajjan RA, ten Berg J, Cattaneo M (2015). Antithrombotic therapy in the elderly: expert position paper of the European Society of Cardiology Working Group on Thrombosis. *European Heart Journal*.

[4] Capranzano P, Angiolillo DJ (2021). Antithrombotic Management of Elderly Patients with Coronary Artery Disease. *JACC: Cardiovascular Interventions*.

[5] Capodanno D, Angiolillo DJ (2010). Antithrombotic therapy in the elderly. *Journal of the American College of Cardiology*.

[6] Maree AO, Curtin RJ, Dooley M, Conroy RM, Crean P, Cox D (2005). Platelet response to low-dose enteric-coated aspirin in patients with stable cardiovascular disease. *Journal of the American College of Cardiology*.

[7] Rocca B, Husted S (2016). Safety of Antithrombotic Agents in Elderly Patients with Acute Coronary Syndromes. *Drugs & Aging*.

[8] Ferreiro JL, Vivas D, De La Hera JM, Marcano AL, Lugo LM, Gómez-Polo JC (2019). High and low on-treatment platelet reactivity to P2Y12 inhibitors in a contemporary cohort of acute coronary syndrome patients undergoing percutaneous coronary intervention. *Thrombosis Research*.

[9] Silvain J, Cayla G, Hulot JS, Finzi J, Kerneis M, O’Connor SA (2012). High on-thienopyridine platelet reactivity in elderly coronary patients: the SENIOR-PLATELET study. *European Heart Journal*.

[10] Gimbel M, Qaderdan K, Willemsen L, Hermanides R, Bergmeijer T, de Vrey E (2020). Clopidogrel versus ticagrelor or prasugrel in patients aged 70 years or older with non-ST-elevation acute coronary syndrome (POPular AGE): the randomised, open-label, non-inferiority trial. *The Lancet*.

[11] Wiviott SD, Braunwald E, McCabe CH, Montalescot G, Ruzyllo W, Gottlieb S (2007). Prasugrel versus clopidogrel in patients with acute coronary syndromes. *The New England Journal of Medicine*.

[12] Wrishko RE, Ernest CS, Small DS, Li YG, Weerakkody GJ, Riesmeyer JR (2009). Population pharmacokinetic analyses to evaluate the influence of intrinsic and extrinsic factors on exposure of prasugrel active metabolite in TRITON-TIMI 38. *Journal of Clinical Pharmacology*.

[13] Erlinge D, Gurbel PA, James S, Lindahl TL, Svensson P, Ten Berg JM (2013). Prasugrel 5 mg in the very elderly attenuates platelet inhibition but maintains noninferiority to prasugrel 10 mg in nonelderly patients: the GENERATIONS trial, a pharmacodynamic and pharmacokinetic study in stable coronary artery disease patients. *Journal of the American College of Cardiology*.

[14] Savonitto S, Ferri LA, Piatti L, Grosseto D, Piovaccari G, Morici N (2018). Comparison of Reduced-Dose Prasugrel and Standard-Dose Clopidogrel in Elderly Patients with Acute Coronary Syndromes Undergoing Early Percutaneous Revascularization. *Circulation*.

[15] Wallentin L, Becker RC, Budaj A, Cannon CP, Emanuelsson H, Held C (2009). Ticagrelor versus clopidogrel in patients with acute coronary syndromes. *The New England Journal of Medicine*.

[16] Cayla G, Cuisset T, Silvain J, Leclercq F, Manzo-Silberman S, Saint-Etienne C (2016). Platelet function monitoring to adjust antiplatelet therapy in elderly patients stented for an acute coronary syndrome (ANTARCTIC): an open-label, blinded-endpoint, randomised controlled superiority trial. *The Lancet*.

[17] Boersma E, Harrington RA, Moliterno DJ, White H, Théroux P, Van de Werf F (2002). Platelet glycoprotein IIb/IIIa inhibitors in acute coronary syndromes: a meta-analysis of all major randomised clinical trials. *The Lancet*.

[18] Marcano AL, Ferreiro JL (2016). Role of New Antiplatelet Drugs on Cardiovascular Disease: Update on Cangrelor. *Current Atherosclerosis Reports*.

[19] Caldeira D, Nunes-Ferreira A, Rodrigues R, Vicente E, Pinto FJ, Ferreira JJ (2019). Non-vitamin K antagonist oral anticoagulants in elderly patients with atrial fibrillation: A systematic review with meta-analysis and trial sequential analysis. *Archives of Gerontology and Geriatrics*.

[20] Bonanad C, García-Blas S, Torres Llergo J, Fernández-Olmo R, Díez-Villanueva P, Ariza-Solé A (2021). Direct Oral Anticoagulants versus Warfarin in Octogenarians with Nonvalvular Atrial Fibrillation: A Systematic Review and Meta-Analysis. *Journal of Clinical Medicine*.

[21] Martínez-Sellés M, Bayés de Luna A (2017). Atrial fibrillation in the elderly. *Journal of Geriatric Cardiology*.

[22] Ruiz Ortiz M, Muñiz J, Raña Míguez P, Roldán I, Marín F, Asunción Esteve-Pastor M (2018). Inappropriate doses of direct oral anticoagulants in real-world clinical practice: prevalence and associated factors. A subanalysis of the FANTASIIA Registry. *Europace*.

[23] Esteve-Pastor MA, Martín E, Alegre O, Formiga F, Sanchís J, López-Palop R (2021). Impact of frailty and atrial fibrillation in elderly patients with acute coronary syndromes. *European Journal of Clinical Investigation*.

[24] Guerrero C, Ariza-Solé A, Formiga F, Martínez-Sellés M, Vidán MT, Aboal J (2018). Applicability of the PRECISE-DAPT score in elderly patients with myocardial infarction. *Journal of Geriatric Cardiology*.

[25] Dewilde WJM, Oirbans T, Verheugt FWA, Kelder JC, De Smet BJGL, Herrman JP (2013). Use of clopidogrel with or without aspirin in patients taking oral anticoagulant therapy and undergoing percutaneous coronary intervention: an open-label, randomised, controlled trial. *The Lancet*.

[26] García-Blas S, Cordero A, Diez-Villanueva P, Martinez-Avial M, Ayesta A, Ariza-Solé A (2021). Acute Coronary Syndrome in the Older Patient. *Journal of Clinical Medicine*.

[27] Bonanad C, Esteve-Claramunt F, García-Blas S, Ayesta A, Díez-Villanueva P, Pérez-Rivera JÁ (2022). Antithrombotic Therapy in Elderly Patients with Acute Coronary Syndromes. *Journal of Clinical Medicine*.

[28] Zwart B, Bor WL, de Veer AJWM, Mahmoodi BK, Kelder JC, Lip GYH (2022). A novel risk score to identify the need for triple antithrombotic therapy in patients with atrial fibrillation undergoing percutaneous coronary intervention: a post hoc analysis of the RE-DUAL PCI trial. *EuroIntervention*.

[29] Hindricks G, Potpara T, Dagres N, Arbelo E, Bax JJ, Blomström-Lundqvist C (2021). 2020 ESC Guidelines for the diagnosis and management of atrial fibrillation developed in collaboration with the European Association for Cardio-Thoracic Surgery (EACTS): The Task Force for the diagnosis and management of atrial fibrillation of the European Society of Cardiology (ESC) Developed with the special contribution of the European Heart Rhythm Association (EHRA) of the ESC. *European Heart Journal*.

[30] Damluji AA, Forman DE, Wang TY, Chikwe J, Kunadian V, Rich MW (2023). Management of Acute Coronary Syndrome in the Older Adult Population: A Scientific Statement from the American Heart Association. *Circulation*.

[31] Costa F, van Klaveren D, James S, Heg D, Räber L, Feres F (2017). Derivation and validation of the predicting bleeding complications in patients undergoing stent implantation and subsequent dual antiplatelet therapy (PRECISE-DAPT) score: a pooled analysis of individual-patient datasets from clinical trials. *The Lancet*.

[32] Yeh RW, Secemsky EA, Kereiakes DJ, Normand SLT, Gershlick AH, Cohen DJ (2016). Development and Validation of a Prediction Rule for Benefit and Harm of Dual Antiplatelet Therapy Beyond 1 Year After Percutaneous Coronary Intervention. *The Journal of the American Medical Association*.

[33] Díez-Villanueva P, Arizá-Solé A, Vidán MT, Bonanad C, Formiga F, Sanchis J (2019). Recommendations of the Geriatric Cardiology Section of the Spanish Society of Cardiology for the Assessment of Frailty in Elderly Patients with Heart Disease. *Revista Espanola De Cardiologia*.

[34] Kwok CS, Achenbach S, Curzen N, Fischman DL, Savage M, Bagur R (2020). Relation of Frailty to Outcomes in Percutaneous Coronary Intervention. *Cardiovascular Revascularization Medicine*.

[35] Kurobe M, Uchida Y, Ishii H, Yamashita D, Yonekawa J, Satake A (2021). Impact of the clinical frailty scale on clinical outcomes and bleeding events in patients with ST-segment elevation myocardial infarction. *Heart and Vessels*.

[36] Ariza-Solé A, Guerrero C, Formiga F, Aboal J, Abu-Assi E, Marín F (2018). Global Geriatric Assessment and In-Hospital Bleeding Risk in Elderly Patients with Acute Coronary Syndromes: Insights from the LONGEVO-SCA Registry. *Thrombosis and Haemostasis*.

[37] Ariza-Solé A, Formiga F, Bardají A, Viana-Tejedor A, Alegre O, de Frutos F (2019). Clinical Characteristics and Prognosis of Very Elderly Patients with Acute Coronary Syndrome Treated with Ticagrelor: insights from the LONGEVO-SCA Registry. *Revista Espanola De Cardiologia*.

[38] Pavasini R, Maietti E, Tonet E, Bugani G, Tebaldi M, Biscaglia S (2019). Bleeding Risk Scores and Scales of Frailty for the Prediction of Haemorrhagic Events in Older Adults with Acute Coronary Syndrome: Insights from the FRASER study. *Cardiovascular Drugs and Therapy*.

[39] Faggioni M, Baber U, Sartori S, Chandrasekhar J, Cohen DJ, Henry TD (2019). Influence of Baseline Anemia on Dual Antiplatelet Therapy Cessation and Risk of Adverse Events After Percutaneous Coronary Intervention. *Circulation: Cardiovascular Interventions*.

[40] Lucà F, Parrini I, Abrignani MG, Rao CM, Piccioni L, Di Fusco SA (2022). Management of Acute Coronary Syndrome in Cancer Patients: It’s High Time We Dealt with It. *Journal of Clinical Medicine*.

[41] Díez-Villanueva P, García-Acuña JM, Raposeiras-Roubin S, Barrabés JA, Cordero A, Martínez-Sellés M (2021). Prognosis Impact of Diabetes in Elderly Women and Men with Non-ST Elevation Acute Coronary Syndrome. *Journal of Clinical Medicine*.

[42] Vicent L, Ariza-Solé A, Alegre O, Sanchís J, López-Palop R, Formiga F (2019). Octogenarian women with acute coronary syndrome present frailty and readmissions more frequently than men. *European Heart Journal. Acute Cardiovascular Care*.

[43] Ayesta A, Bonanad C, Díez-Villanueva P, García-Blas S, Ariza-Solé A, Martínez-Sellés M (2022). Ethical considerations in elderly patients with acute coronary syndrome. *Reviews in Cardiovascular Medicine*.

[44] Walker DM, Gale CP, Lip G, Martin-Sanchez FJ, McIntyre HF, Mueller C (2018). Editor’s Choice - Frailty and the management of patients with acute cardiovascular disease: A position paper from the Acute Cardiovascular Care Association. *European Heart Journal: Acute Cardiovascular Care*.

[45] Sanchis J, Bonanad C, Ruiz V, Fernández J, García-Blas S, Mainar L (2014). Frailty and other geriatric conditions for risk stratification of older patients with acute coronary syndrome. *American Heart Journal*.

[46] Sanchis J, Ruiz V, Bonanad C, Valero E, Ruescas-Nicolau MA, Ezzatvar Y (2017). Prognostic Value of Geriatric Conditions Beyond Age After Acute Coronary Syndrome. *Mayo Clinic Proceedings*.

[47] Sánchez E, Vidán MT, Serra JA, Fernández-Avilés F, Bueno H (2011). Prevalence of geriatric syndromes and impact on clinical and functional outcomes in older patients with acute cardiac diseases. *Heart*.

[48] Alegre O, Formiga F, López-Palop R, Marín F, Vidán MT, Martínez-Sellés M (2018). An Easy Assessment of Frailty at Baseline Independently Predicts Prognosis in Very Elderly Patients With Acute Coronary Syndromes. *Journal of the American Medical Directors Association*.

[49] Rodríguez-Queraltó O, Formiga F, López-Palop R, Marín F, Vidán MT, Martínez-Sellés M (2020). FRAIL Scale also Predicts Long-Term Outcomes in Older Patients With Acute Coronary Syndromes. *Journal of the American Medical Directors Association*.

[50] Ekerstad N, Javadzadeh D, Alexander KP, Bergström O, Eurenius L, Fredrikson M (2022). Clinical Frailty Scale classes are independently associated with 6-month mortality for patients after acute myocardial infarction. *European Heart Journal: Acute Cardiovascular Care*.

[51] Tegn N, Abdelnoor M, Aaberge L, Endresen K, Smith P, Aakhus S (2016). Invasive versus conservative strategy in patients aged 80 years or older with non-ST-elevation myocardial infarction or unstable angina pectoris (After Eighty study): an open-label randomised controlled trial. *The Lancet*.

[52] Sanchis J, Núñez E, Barrabés JA, Marín F, Consuegra-Sánchez L, Ventura S (2016). Randomized comparison between the invasive and conservative strategies in comorbid elderly patients with non-ST elevation myocardial infarction. *European Journal of Internal Medicine*.

[53] Sanchis J, García Acuña JM, Raposeiras S, Barrabés JA, Cordero A, Martínez-Sellés M (2021). Comorbidity burden and revascularization benefit in elderly patients with acute coronary syndrome. *Revista Espanola De Cardiologia*.

[54] Cepas-Guillén PL, Echarte-Morales J, Caldentey G, Gómez EM, Flores-Umanzor E, Borrego-Rodriguez J (2022). Outcomes of Nonagenarians With Acute Coronary Syndrome. *Journal of the American Medical Directors Association*.

[55] Núñez J, Ruiz V, Bonanad C, Miñana G, García-Blas S, Valero E (2017). Percutaneous coronary intervention and recurrent hospitalizations in elderly patients with non ST-segment acute coronary syndrome: The role of frailty. *International Journal of Cardiology*.

[56] Llaó I, Ariza-Solé A, Sanchis J, Alegre O, López-Palop R, Formiga F (2018). Invasive strategy and frailty in very elderly patients with acute coronary syndromes. *EuroIntervention*.

[57] Sanchis J, Ariza-Solé A, Abu-Assi E, Alegre O, Alfonso F, Barrabés JA (2019). Invasive Versus Conservative Strategy in Frail Patients with NSTEMI: The MOSCA-FRAIL Clinical Trial Study Design. *Revista Espanola De Cardiologia*.

[58] Sanchis J, Bueno H, Miñana G, Guerrero C, Martí D, Martínez-Sellés M (2023). Effect of Routine Invasive vs Conservative Strategy in Older Adults With Frailty and Non-ST-Segment Elevation Acute Myocardial Infarction: A Randomized Clinical Trial. *JAMA Internal Medicine*.

[59] Majithia A, Bhatt DL (2019). Novel Antiplatelet Therapies for Atherothrombotic Diseases. *Arteriosclerosis, Thrombosis, and Vascular Biology*.

[60] Valgimigli M, Gragnano F, Branca M, Franzone A, Baber U, Jang Y (2021). P2Y12 inhibitor monotherapy or dual antiplatelet therapy after coronary revascularisation: individual patient level meta-analysis of randomised controlled trials. *British Medical Journal*.

[61] Valgimigli M, Frigoli E, Heg D, Tijssen J, Jüni P, Vranckx P (2021). Dual Antiplatelet Therapy after PCI in Patients at High Bleeding Risk. *The New England Journal of Medicine*.

[62] Sørensen R, Hansen ML, Abildstrom SZ, Hvelplund A, Andersson C, Jørgensen C (2009). Risk of bleeding in patients with acute myocardial infarction treated with different combinations of aspirin, clopidogrel, and vitamin K antagonists in Denmark: a retrospective analysis of nationwide registry data. *The Lancet*.

[63] Piccini JP, Caso V, Connolly SJ, Fox KAA, Oldgren J, Jones WS (2022). Safety of the oral factor XIa inhibitor asundexian compared with apixaban in patients with atrial fibrillation (PACIFIC-AF): a multicentre, randomised, double-blind, double-dummy, dose-finding phase 2 study. *The Lancet*.

[64] Rao SV, Kirsch B, Bhatt DL, Budaj A, Coppolecchia R, Eikelboom J (2022). A Multicenter, Phase 2, Randomized, Placebo-Controlled, Double-Blind, Parallel-Group, Dose-Finding Trial of the Oral Factor XIa Inhibitor Asundexian to Prevent Adverse Cardiovascular Outcomes After Acute Myocardial Infarction. *Circulation*.

[65] Castellano JM, Pocock SJ, Bhatt DL, Quesada AJ, Owen R, Fernandez-Ortiz A (2022). Polypill Strategy in Secondary Cardiovascular Prevention. *The New England Journal of Medicine*.

[66] Braunwald E (2022). How to live to 100 before developing clinical coronary artery disease: a suggestion. *European Heart Journal*.

[67] Giallauria F, Di Lorenzo A, Venturini E, Pacileo M, D’Andrea A, Garofalo U (2021). Frailty in Acute and Chronic Coronary Syndrome Patients Entering Cardiac Rehabilitation. *Journal of Clinical Medicine*.

